# Hydroxyapatite Formation on Coated Titanium Implants Submerged in Simulated Body Fluid

**DOI:** 10.3390/ma13245593

**Published:** 2020-12-08

**Authors:** Tatiana Aviles, Shu-Min Hsu, Arthur Clark, Fan Ren, Chaker Fares, Patrick H. Carey, Josephine F. Esquivel-Upshaw

**Affiliations:** 1 Department of Restorative Dental Sciences, Division of Prosthodontics, College of Dentistry, University of Florida, Gainesville, FL 32610, USA; aviles.tmarie@ufl.edu (T.A.); shuminhsu@ufl.edu (S.-M.H.); BCLARK@dental.ufl.edu (A.C.); 2 Department of Chemical Engineering, Herbert Wertheim College of Engineering, University of Florida, Gainesville, FL 32611, USA; fren@che.ufl.edu (F.R.); c.fares@ufl.edu (C.F.); careyph@ufl.edu (P.H.C.IV)

**Keywords:** hydroxyapatite, simulated body fluid, dental implants

## Abstract

Titanium implants are commonly used in the field of dentistry for prosthetics such as crowns, bridges, and dentures. For successful therapy, an implant must bind to the surrounding bone in a process known as osseointegration. The objective for this ongoing study is to determine the potential of different implant surface coatings in providing the formation of hydroxyapatite (HA). The coatings include titanium nitride (TiN), silicon dioxide (SiO_2_), and quaternized titanium nitride (QTiN). The controls were a sodium hydroxide treated group, which functioned as a positive control, and an uncoated titanium group. Each coated disc was submerged in simulated body fluid (SBF), replenished every 48 h, over a period of 28 days. Each coating successfully developed a layer of HA, which was calculated through mass comparisons and observed using scanning electron microscopy (SEM) and energy dispersive analysis x-rays (EDX). Among these coatings, the quaternized titanium nitride coating seemed to have a better yield of HA. Further studies to expand the data concerning this experiment are underway.

## 1. Introduction

Dental implants in a variety of forms have appeared as early as 2500BC among the ancient Egyptians, with the introduction of titanium implants emerging in the 1970s [1]. Today, most dental implants are made of titanium or titanium alloys (Ti6Al4V) because of this metal’s mechanical strength, low density, corrosion resistance and high biocompatibility [2,3,4,5]. Titanium has the unique ability to integrate with bone in a process called osseointegration [5]. Osseointegration is the binding of bone to the implant site through a complex matrix of osteogenic cells, platelets, and blood clots [6]. This may be due to titanium’s hydrophilic exterior, surface grooves, or chemical nature that induces bone fusion with the implant, making restoration more stable and longer lasting [7]. With continual modifications, titanium can be improved to address areas of concern, such as limiting inflammation around an implant site, making the metal highly favorable and widely used in bone-implant technology [8].

When appropriately modified, titanium implants can elicit preliminary apatite growth on their surface and bond to living bone when subjected to bodily conditions [9]. In these early stages of bone growth, mineralizing cells including osteoblasts generate nano-sized HA which forms a matrix of vesicles to enable bone formation to progress [10]. Hydroxyapatite or HA (Ca_5_(PO_4_)_3_OH) is an inorganic calcium and phosphorous-containing component making up 70% of human bone content, including enamel [11]. HA has various biomedical applications due to HA’s biocompatibility and highly porous structure [12]. Within dentistry alone, research studies on HA are demonstrating this material’s potential in a variety of applications including implant coatings to facilitate osseointegration, as a bone substitute in intraosseous implantation, treatment of dentin hypersensitivity, and repair of microscopic defects resulting from bleaching agents [13,14]. Another successful application of HA is in glass ionomer cements specifically used in pediatric procedures to treat dental caries [15]. However, despite the large number of studies advocating the use of HA, other studies have demonstrated some disadvantages with use of this material [16]. On a nanoscale, one concern is whether defects that may appear when doping can cause impurities, which lead to cytotoxicity and eventual material rejection in an organism [16]. Another study claims that hydroxyapatite-protein-composite, included as an active ingredient in some toothpastes, correlates to the development of several chronic diseases [14]. Collectively, HA should be viewed as a subject of study and require further explanation when regarding the findings of this study.

Research on modifying an implant’s surface to yield more successful osseointegration is constantly being undertaken. This may include pretreatments to remove contaminants, mechanical treatments (polishing, sandblasting), physical treatments (plasma or thermal spray), chemical treatments (chemical etching, SBF immersion), electrochemical treatments (anodic oxidation, UV photocatalysis) or simple coatings (e.g., calcium phosphate, silicon carbide, or HA) [17,18,19]. Studies regarding surface modifications of titanium suggest that micro-rough and porous surfaces improve HA formation and bone growth [20,21]. Apart from surface topology, chemical composition and surface energy have also been reported to influence bone formation processes [7]. The following research will include some of the surface modifications listed above to promote the osseointegrative potential of HA. 

Within this experiment, simulated body fluid (SBF) was used as a medium for HA formation to occur. Under SBF conditions, additional modifications and biomimetic agents can be consolidated into the HA growth [22]. As far as the authors know, there are studies concerning the plasma-spraying of HA onto implants as a coating, but there are limited published reports on the capacity of the coatings themselves to promote hydroxyapatite formation under SBF conditions. The objective of this study is to investigate the HA formation ability of different thin film coatings in vivo through immersion in simulated body fluid in vitro as a function of the quantity of HA that forms on these coatings, which have anti-corrosive and anti-bacterial properties [23,24,25,26]. This study hypothesizes that all coatings will form similar amounts of HA on their surfaces when exposed to SBF for a 28-day period of immersion. NaOH is supported by experimental findings to have the most success in developing a layer of HA. The observations made from this study will help determine which of the coated samples, other than the NaOH used as a control, will yield the highest quantity of HA to be studied in further experiments. 

## 2. Materials and Methods

### 2.1. Experimental Design

There were five groups in this study (i) non-coated titanium disk as reference, (ii) NaOH treated disks as a positive control, (iii) SiO_2_ coated disks, (iv) TiN coated disks and (v) quaternized TiN coated disks. Two disks were used per each group. All the discs were placed into round-bottom tubes (one disc per tube) and suspended in SBF for a period of 28 days. Each vial was placed in a water bath that maintained a temperature of 37 °C throughout the 28-day duration. The SBF in each vial was replaced every 48 h. Each vial had 10 mL of SBF added and was left undisturbed except for when the liquid was replenished. After the 28-day period, the discs were rinsed with deionized water and dried at room temperature for 24–48 h prior to being weighed and scanned again.

### 2.2. Ti Disc Preparation

9–10 mm long cylinders made from 99.9% titanium were cut into 3 mm ± 0.5 mm thick discs with a 4 mm diameter using a wafering blade (IsoMet 15HC, Buehler, Gainesville, FL, USA) with an average of 100 g of added weight and a blade speed that increased to 900 rpm at max speed. The blade was lubricated with Buehler Cool 2 Fluid, recommended for most applications. Each cut disc was then progressively polished under a stream of water (EcoMet 250/300, Buehler, Gainesville, FL, USA), with a base speed of 110 rpm and a head speed of 60 rpm in opposite spin directions. In addition, 1.8–2.7 kgs of pressure were applied per disc. For the purpose of this experiment, only one side was polished, first using 320 grit and then using 600 grit. The purpose for polishing the samples was to create consistent micro-smoothness on the surface of each disc to promote uniform HA growth across all samples. To hold the discs in place, all samples were glued onto the polishing head. Any glue residues were later removed by soaking in ethanol for 24 h and then rinsed with deionized water. Prior to coating, polished sample surfaces were scanned for reference using an optical microscope. Afterwards, discs were coated with SiO_2_, TiN, and QTiN. Each coating had two discs for this pilot study to determine differences between the coatings. The two discs per coating were submerged separately in test tubes full of SBF for a total of ten discs that were submerged and observed.

### 2.3. Sample Coating

For the purpose of this experiment, the differences of HA formation on samples coated with SiO_2_, TiN, and QTiN were tested. SiO_2_ was chosen as a coating because of this material’s osteoinductive properties as silicon, being an essential element in bone, is released during hydrolytic degradation of the coating compound [2]. Plasma-Enhanced Chemical Vapor Deposition (PECVD; PlasmaTherm 790, Saint Petersburg, FL, USA) was used for silicon dioxide formation. The precursors of silane/nitrous oxide were applied during the silicon dioxide deposition process. TiN coatings have shown improved interaction with living tissue and bone regeneration regarding implants [27]. TiN has high strength, biocompatibility, and corrosion-resistance, which makes TiN a good coating for dental implants [27]. The TiN coating was deposited using rf-magnetron sputtering to create a layer several microns thick [23]. Equally durable, QTiN was also tested on the basis that this coating is more antimicrobial than TiN alone. Quaternization produces quaternary charged surface nitrogen atoms, N+, which has been shown to induce cell wall destruction and apoptosis of bacteria [23]. Considering that peri-implantitis is caused by bacteria, molecules with quaternary nitrogen atoms can reduce bacterial counts [23]. The Menschutkin reaction, which uses allyl bromide deposited onto the TiN coating, was utilized to convert the nitrogen into quaternary nitrogen for the quaternized TiN coating.

The control groups included uncoated Ti discs as well as discs treated with NaOH. The uncoated discs were polished to 600 grit with no additional surface or chemical modifications to determine how titanium’s qualities alone would react under SBF conditions in HA formation. Alkali-treated implant surfaces have been observed to be a conducive treatment for HA formation in SBF in vitro studies [28]. The alkalinity from the hydroxyl groups of NaOH work to activate the titanium disc surface [22]. These samples were submerged in 5 M NaOH at 60 °C for 24 h [29]. The duration of immersion and concentration of NaOH affect sodium titanate development, and consequentially, the efficiency in which the titanium implant and surrounding bone interact during preliminary healing stages [28].

### 2.4. Simulated Body Fluid (SBF) Preparation

SBF was made to have ion concentrations like that of human blood plasma as shown in Table 1 and was buffered to a pH of 7.4. A beaker containing the mix was placed in a water bath at a temperature of 36.5–37.0 °C. A magnetic stirrer plate was used to mix the solution over 2 h while the listed compounds were added in the order listed in Table 2. Note that the original concentrations were for a 1 L solution and were adjusted to produce a 1.5 L solution of SBF. The components of the SBF were added slowly to avoid a cloudy final solution; the goal was to have clear SBF as a finished product. The pH was brought to 7.4 by varying hydrochloric acid and tris(hydroxymethyl)aminomethane accordingly. The HCl was taken from a stock of 37% HCl. A pH electrode was used to monitor the changes in pH as the buffer was made to match in vitro conditions.

### 2.5. Hydroxyapatite Formation under a Digital Microscope System

After SBF immersion for 28 days, the surfaces of each sample were photographed at 20 × 30, 20 × 100, and 20 × 200 magnification (Digital Microscope System, Keyence, Gainesville, FL, USA) before and after submersion in SBF. Discs were centered under the lens and multiple images were taken at each magnification of the surface and edges of each disc. 

### 2.6. Scanning Electron Microscopy (SEM) Imaging

The SEM (Mira3, Tescan, Gainesville, FL, USA) was used to further investigate formation and morphologies of HA development. SEM conditions includes a working distance of 13.10–15.06, beam intensity of 15, and magnification of 79–81× and 13.0–15.06×. Samples were coated with 10 nm of carbon prior to SEM scanning. 

### 2.7. Energy Dispersive x-Rays Analysis (EDX)

EDX (FEI Helios NanoLab 600, ThermoFisher, Gainesville, FL, USA) was used to determine the surface composition of the hydroxyapatite layer. The conditions used included a kV of 10, amp time of 7.68, live time of 150, takeoff angle of 32–34, resolution of 122.8. The ions of interest were the main components of hydroxyapatite: calcium, phosphorous, and oxygen. More specifically, OH^−^, PO_4_^3−^, and Ca^2+^ were of interest considering that apatite formation depends on these ions. The average percent error for each ion includes oxygen at 9.51%, phosphorus at 3.36%, and calcium at 3.56%.

## 3. Results

### 3.1. Optical Magnification

**Figure 1:**
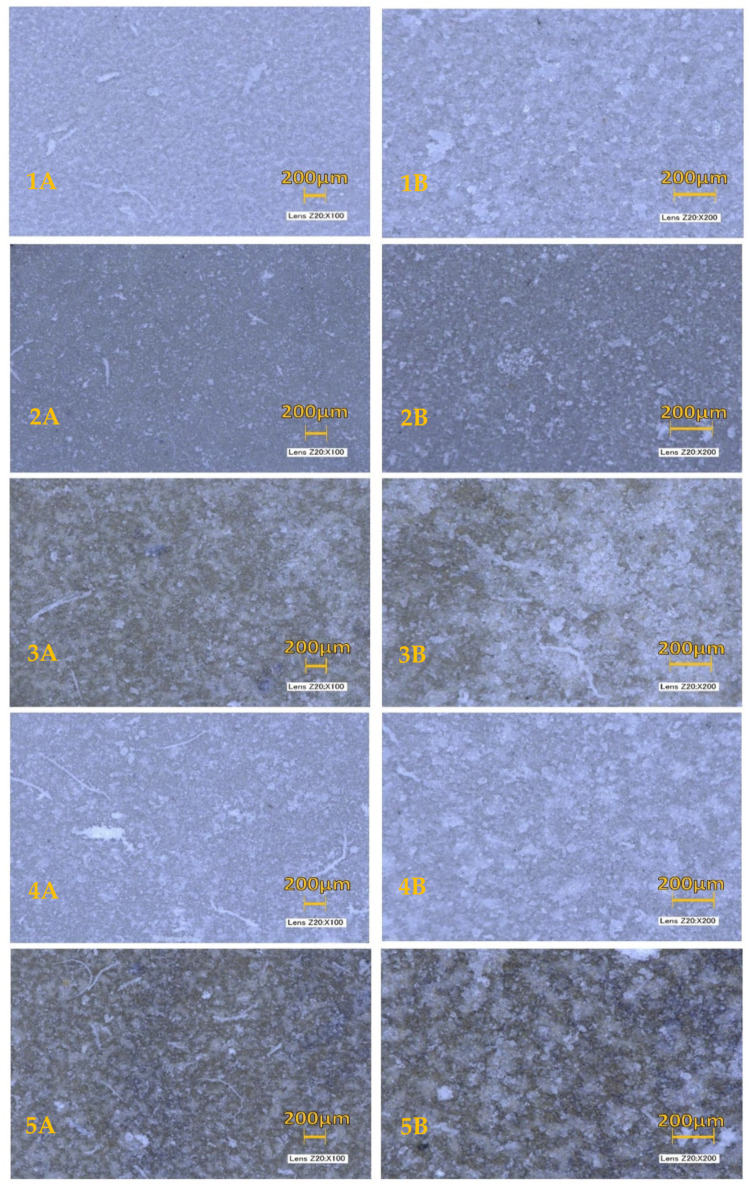
Optical Microscope HA Formation Comparisons. Magnification seen at 20 × 100 (**A**) and 20 × 200 (**B**): (**1**) control titanium; (**2**) sodium hydroxide coating; (**3**) quaternized titanium nitride coating; (**4**) silicon dioxide coating; (**5**) titanium nitride coating.

All sample surfaces were scanned using a digital optical microscope (Digital Microscope System, Keyence, Gainesville, FL, USA) preceding immersion in the simulated body fluid. After immersion, coated samples were also viewed under an optical microscope to determine if HA growth had occurred after the 28-day growing period. As is seen in Figure 1, each sample had a layer of growth on the top and sides of the disc.

### 3.2. Scanning Electron Microscopy

**Figure 2:**
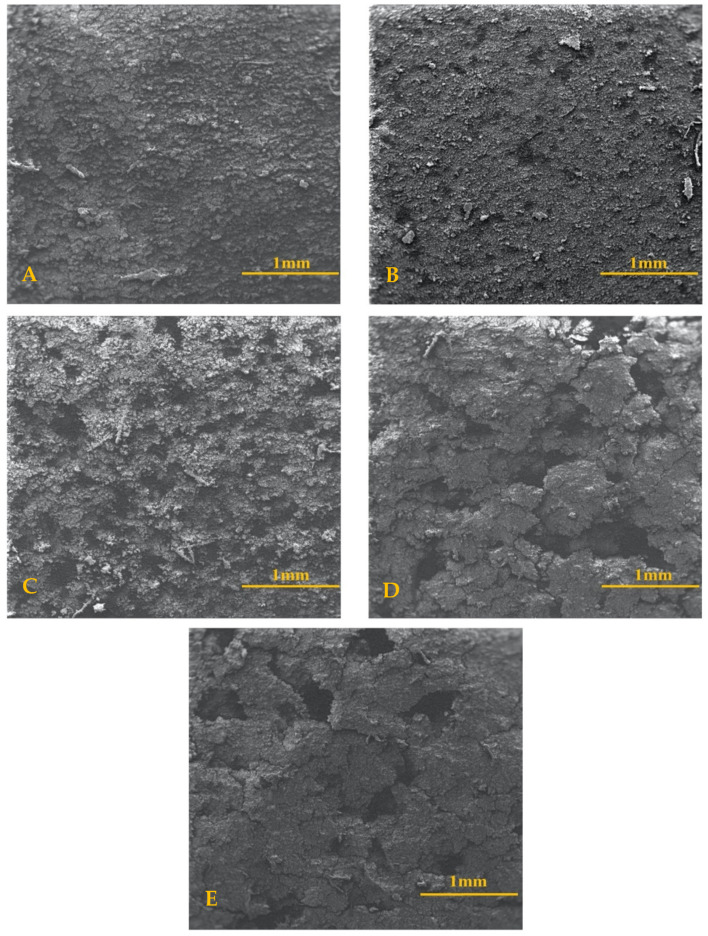
SEM of HA Formation Comparisons (1 mm scale). Scanning electron microscopy images taken at a field view of 3.40–3.50 mm with a 1 mm scale for (**A**) control, (**B**) sodium hydroxide, (**C**) quaternized titanium nitride, (**D**) silicon dioxide, (**E**) titanium nitride.

Scanning electron microscopy showed a variety of topographies across sample coatings. In the larger field-view (3.4 mm) captured in Figure 2, the control and NaOH treated discs maintain a sandpaper-like appearance in comparably small bumps along the surface of the sample. The SiO_2_ and TiN treated discs had larger groupings of HA structures and exhibited the traits of wet or cracking sand. The sample with a coating of QTiN appears to share similar traits as those mentioned above, with clusters slightly larger than sandpaper but smaller than the groupings found on both SiO_2_ and TiN coatings.

**Figure 3:**
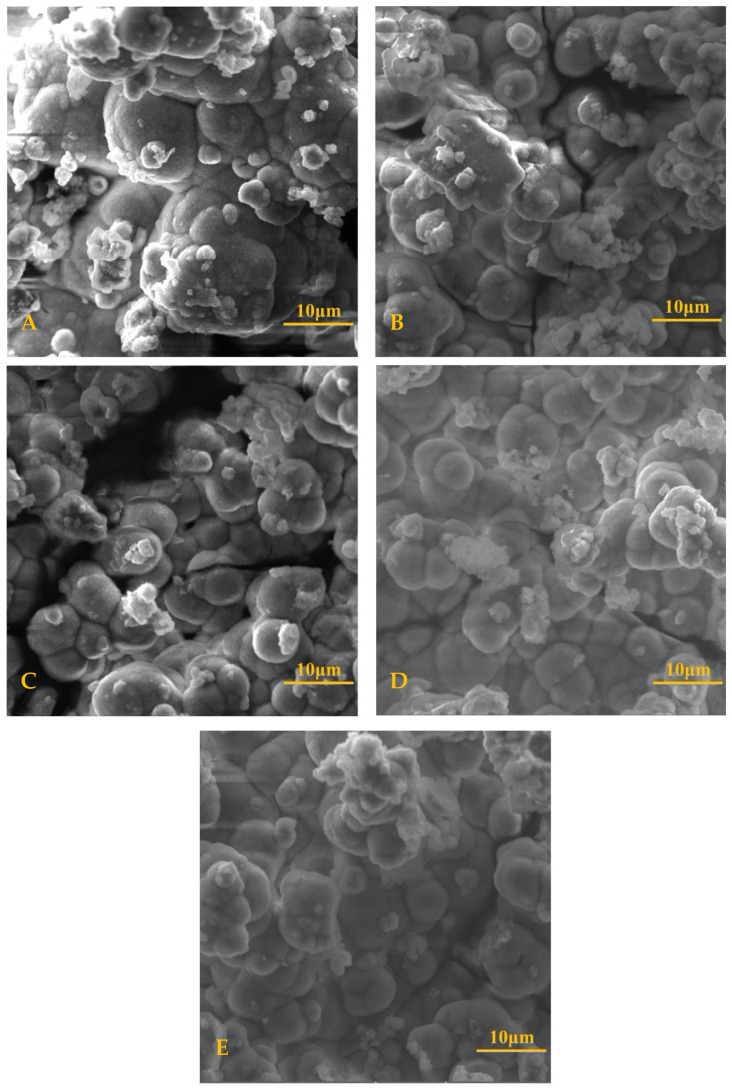
SEM of HA Formation Comparison (10 μm scale). Scanning electron microscopy images taken at a field view of 50.0 μm with a 10 μm scale for (**A**) control; (**B**) sodium hydroxide; (**C**) quaternized titanium nitride; (**D**) silicon dioxide; (**E**) titanium nitride.

HA formation maintained a lattice of bulbous shapes with thicker, more clustered regions when seen from a 50 μm × 50 μm field view. This magnification displayed uniform growth of HA and no titanium from the disc surface could be seen through the HA layer. Differences in shape and size were difficult to distinguish at higher magnifications between samples as can be seen in Figure 3.

### 3.3. Energy Dispersive Analysis X-rays (EDX)

**Figure 4:**
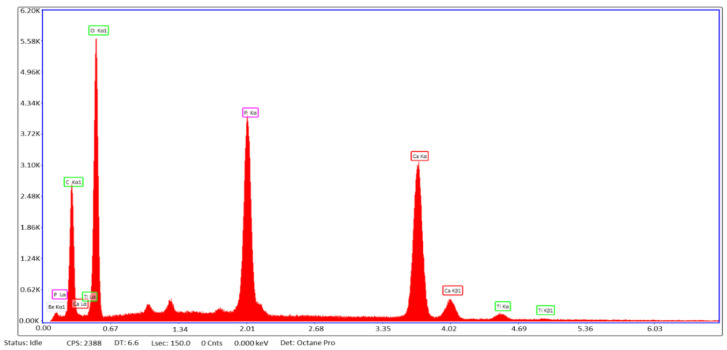
Representative EDX. Energy dispersive analysis x-ray of the quaternized titanium nitride sample is representative of what all the sample EDX graphs looked like.

The results determined from the energy dispersive analysis x-ray were almost identical across all coatings. Figure 4 is a representative EDX of all the coatings tested because the samples maintained very similar peaks in their own readings. Table 3 lists the ions of interest that compose HA (oxygen, phosphorous, calcium) and the slight variations in atomic percentages across all coatings. Most important was the ratio of calcium to phosphorous since HA has a stochiometric ratio of calcium to phosphate of 1.67 [31]. The results calculated were similar across the coatings with SiO_2_ at 1.86, TiN at 1.83, QTiN at 1.84, and the controls of NaOH at 1.81 and Ti at 1.92. Table 3 can be reviewed for further atomic percentages of oxygen, phosphorus, and calcium individually. By submerging the discs in SBF over a period of 28-days, the formation of hydroxyapatite on all samples was encouraged.

### 3.4. Thickness of Hydroxyapatite Layer

As mentioned, all samples were weighed prior to and after submersion in SBF. The differences in weight are listed in Table 4 and were measured using a standard analytical balance (AS 60/220.R2 PLUS analytical balance; RADWAG; Gainesville, FL, USA) to ± 0.01 mg. The difference in mass before and after submersion determined the amount of HA that accumulated on each disc over the duration of submersion. The diameter and height of the discs were determined using a 0–150 mm digital vernier caliper, later converted to µm. These measurements were used to determine the surface area of the disc (Table 4) and considering the density of hydroxyapatite (3.16 g/cm^3^), the approximate thickness of each HA layer was calculated as well [32]. The values of approximate HA thickness and mass are listed in Table 4.

## 4. Discussion

Hydroxyapatite has the ability to facilitate osseointegration [12,33]. All the coatings successfully developed HA on the titanium surface. As seen in implants treated with NaOH, HA was formed in simulated body fluid to facilitate titanium bonding to bone by forming sodium titanate on the implant surface [34]. SBF allows for mineralization of a metal surface which develops a positive charge by interacting with the Ca^2+^ in solution [35]. This enables the titanium surface to absorb PO_4_^3−^ ions and begin HA assembly [35]. In vitro conditions demonstrate that HA forms after 16 h and that osteoblasts are visible after a week or more during the early bone healing process [36,37]. The appearance of HA occurs spontaneously within the Ti-bone interface as a result of the culture medium and blood and may be responsible for the differentiation of stem cells into osteoblasts [36]. 

Numerous factors affect the growth of hydroxyapatite on implant surfaces. In this experiment, the majority of HA seems to have accumulated on the top surface of the discs rather than on the sides, whereas no HA formed on the bottom surface. Scanning electron microscopy of the discs revealed that the HA growth on the sides of the discs was thinner, possibly due to the vertical angle of deposition. Gravity and disc orientation might influence topography and thickness of the HA layer that forms. Furthermore, there might be a limited potential for HA to grow on the underside of each sample [38].

Hydroxyapatite helps osseointegration of the bone to the implant in dental patients, so HA must be reflective of the composition of bone. The EDX results of the study demonstrate high concentrations of phosphorous and calcium levels similar to bone. Table 3 indicates the ratio of calcium to phosphate, which according to the chemical formula of HA, should be roughly 1.67 [31]. Hydroxyapatite is determined to be calcium deficient with a ratio below 1.67 [39,40]. Higher values correlate to HA being formed with lower acidity and solubility [41]. The higher values attained in this experiment may be attributed to the molar ratio of calcium and phosphorous present in the SBF used which has been shown to alter particle size of the HA that forms [40].

Scanning electron microscopy demonstrated that all samples consisted of dense globular structures as a result of the crystal-like growth of HA [42]. Using SEM to determine HA thickness proved difficult due to the lack of distinct parameters as to where the HA layer began and ended. Proper measurements require identical angulation for imaging and a clear set of criteria that identifies where HA growth is contained to determine layer thickness. In live conditions, osteogenic cells and fibroblasts were found to have a greater differentiation and adhesion to surfaces with micro-roughness of 0.2–2 μm [43]. All samples, including the uncoated control, were polished to 600 grit, which may have influenced the nucleation of HA across all the discs in the study. 

The mass and density of HA and disc dimensions were further used to determine the thickness of the HA layer that grew per coating tested. NaOH successfully served as a positive control with the ability to induce HA formation, as stated by the findings of numerous other experiments. As such, NaOH treated discs were used as a positive control. Both control groups, the uncoated disc and the NaOH treated disc, developed the greatest quantity of HA. This may be attributed to the high biocompatibility of the titanium in the untreated control in addition to the micro-roughness provided by the polished surfaces. Treatment of the titanium disc with NaOH results in the formation of an alkali titanate hydrogel layer, which exchanges Na^+^ ions with H_3_O^+^ ions of the SBF [44]. The resulting formation of Ti-OH groups on the sample surface then react with Ca^2+^ and PO_4_^3−^ in the fluid to provide nucleation for hydroxyapatite growth [20]. The neutralization of the positive calcium and negative phosphate charges that form the HA eventually enable the nucleation of bone growth in in vitro conditions [20]. While not performed in this study, heat-exposure of alkali-treated titanium can correlate to increased nucleation and growth of HA due to a more uniform, porous structure [45].

Of the three coatings that were tested, quaternized titanium nitride showed better results in HA formation as a function of thickness and mass of HA, with SiO_2_ and TiN following behind. The potential of SiO_2_ as a coating can be viewed from a biological perspective. As an important component in the formation of bone and cartilage, silicon can incorporate with the HA lattice to encourage osteoblast cell growth and discourage bone resorption at the implant-bone interface [46]. SBF may promote a similar Si-OH layer to form, which neutralizes charged ions in the fluid and allows for HA formation to occur [47]. Regarding titanium nitride, HA growth and cell viability are induced through the partial ionic bonding of TiN which allows for the observation of HA formation under SBF conditions [48]. Taking the additional step of converting the nitrogen to quaternary nitrogen may be responsible for the slightly larger mass and thickness of HA on the QTiN. The quaternary nitrogen makes the coating more positive by increasing the ability to interact with ions in the SBF. The results gathered demonstrate that all coatings are capable of promoting HA formation and could possibly facilitate osseointegration in in vitro conditions. Particularly, the slightly elevated capacity to form HA and the antibacterial properties of QTiN may make this a promising coating for titanium implants.

The experimental design poses limitations to the conclusions which can be made from this study. The HA formation was promoted under SBF conditions where pH and temperature were maintained with little disturbance. In vivo conditions, in contrast, are constantly changing in temperature and pH, which can influence HA formation and osseointegration [26]. Nevertheless, maintaining immersive solutions with a pH of 7.4 has proven to lower levels of HA degradation when compared to samples submerged in a pH of 7.2 or 2.5 which can negatively influence HA morphology [42,49]. Furthermore, a pH of 7.4 is recommended for SBF studies by the International Organization for Standardization (ISO 23317) protocol [42]. Another limitation in the study of these potential coatings in providing optimal HA formation and osseointegration in vivo is that the findings would only apply for an ideally healthy population. Personal habits such as smoking and health conditions such as immune disorder, such as HIV/AIDS (Human Immunodeficiency Virus Infection/Acquired Immunodeficiency Syndrome), have already been studied to decrease implant success, thereby possibly hindering the potential of these coatings during in vivo applications [50].

The data collected in this experiment correlate to other findings regarding the ability of HA to nucleate under SBF conditions and may serve to support studies correlating HA to osseointegrative potential once this research is expanded upon [51]. As a pilot study, further experimentation is required for the clinical implications of the data collected to be conclusive and applicable for treatment. Future emphasis on the growth rate of HA on each coating at different points in time may help determine if the coatings accelerate the initial HA formation and thus shorten the osseointegration process. Possible suspension of the disc and investigation of effects of disc orientation on HA formation should also be considered for the future studies. With the data collected throughout this experiment continuing to serve as a baseline, the main considerations of future studies should be to include: (i) an increased samples size (ii) the effect of gravity and orientation on HA formation, (iii) the rate at which HA formation occurs, and (iv) the corrosive and mechanical properties of coatings for dental implants. 

## 5. Conclusions

Atomic ratio of the ions of interest—those characteristic of hydroxyapatite—were similar across all coatings tested. The presence of high levels of phosphate and calcium indicated hydroxyapatite growth. Using scanning electron microscopy, HA morphology was shown as bulbous and clustered (50 µm field view), although lower magnifications demonstrated slight differences in the overall topology of the HA layer. In a 3.4–3.5 mm field view, the uncoated and NaOH controls had a fine, sandpaper-like appearance, whereas SiO_2_ and TiN had HA formation in larger clumps like wet/cracking sand. QTiN shared characteristics somewhere in between the two extremes explained above.

Over a 28-day growth period, both the untreated and NaOH controls yielded high HA formation as expected. Quaternized titanium nitride had a slightly higher yield of HA than SiO_2_ and TiN. Since this was a pilot study with a relatively small sample size, no conclusion can be made as to which anti-bacterial and anti-corrosive coating was superior in HA growth. Future experiments are planned with an increased sample size that will also consider the effects of external factors such as gravity, period of growth, SBF concentrations, etc., that may affect the quantity of HA observed on each surface.

## 6. Patents

Esquivel-Upshaw JF, Ren F, Clark AE, Batich C, Carey P. Quaternized TiN Anti-Bacterial Coating for Dental Implants. US2019/044556 provisional patent.

## Figures and Tables

**Table 1:** Ion concentration (mM) of SBF and human blood plasma [30].

Ion	SBF	Blood Plasma
Na^+^	142.0	142.0
K^+^	5.0	5.0
Mg^2+^	1.5	1.5
Ca^2+^	2.5	2.5
Cl^−^	148.9	103.0
HCO^3−^	4.2	27.0
HPO_4_^2−^	1.0	1.0
SO_4_^2−^	0.5	0.5

**Table 2:** Reagents for preparing SBF (pH 7.40, 1.5 L) [30].

Order	Reagent	Amount
1	NaCl	12.000 g
2	NaHCO_3_	0.525 g
3	KCl	0.336 g
4	K_2_HPO_4_∙H_2_O	0.342 g
5	MgCl_2_	0.214 g
6	1 M HCl	61.950 mL
7	CaCl_2_∙2H_2_O	0.552 g
8	Na_2_SO_4_	0.107 g
9	(CH_2_OH)_3_CNH_2_	9.086 g

**Table 3:** Atomic percentages (O, P, Ca) from EDX and atomic ratio of Ca/P.

Coating	Atomic %			Ca/P Ratio
	O	P	Ca	Ca/P
Control (Ti)	57.12	14.71	28.17	1.92
NaOH	55.78	15.73	28.49	1.81
QTiN	55.19	15.30	28.12	1.84
SiO_2_	55.12	15.70	29.18	1.86
TiN	57.69	14.96	27.36	1.83

**Table 4:** Hydroxyapatite layer across the samples.

Coating Tested	Disc Surface Area (mm^2^)	HA Mass (mg)	HA Thickness (µm)
Control (Ti)	171.90	2.72	5.04
NaOH	159.05	3.67	7.20
QTiN	101.66	2.18	6.78
SiO_2_	152.07	2.02	4.18
TiN	143.50	2.00	4.44
